# *Lactobacillus fermentum* CRL1446 Ameliorates Oxidative and Metabolic Parameters by Increasing Intestinal Feruloyl Esterase Activity and Modulating Microbiota in Caloric-Restricted Mice

**DOI:** 10.3390/nu8070415

**Published:** 2016-07-07

**Authors:** Matias Russo, Emanuel Fabersani, María C. Abeijón-Mukdsi, Romina Ross, Cecilia Fontana, Alfonso Benítez-Páez, Paola Gauffin-Cano, Roxana B. Medina

**Affiliations:** 1 Centro de Referencia para Lactobacilos (CERELA)-CONICET, Chacabuco 145, San Miguel de Tucumán T4000ILC, Argentina; mrusso@cerela.org.ar (M.R.); faber@cerela.org.ar (E.F.); cabeijon@cerela.org.ar (M.C.A.-M.); 2 Facultad de Ciencias de la Salud, Universidad del Norte Santo Tomás de Aquino, San Miguel de Tucumán T4000IHC, Argentina; gross@fbqf.unt.edu.ar; 3 Universidad Nacional de Tucumán, Facultad de Bioquímica, Química y Farmacia, Ayacucho 471, San Miguel de Tucumán T4000INI, Argentina; 4 Instituto Nacional de Tecnología Agropecuaria INTA-EEA, Ruta Provincial 301 Km 32, Famaillá 4132, Argentina; fontana.cecilia@inta.gob.ar; 5 Microbial Ecology, Nutrition & Health Laboratory, Agrochemistry and Food Technology Institute (IATA-CSIC), Paterna-Valencia 46980, Spain; abenitez@iata.csic.es

**Keywords:** caloric restriction diet, *Lactobacillus fermentum*, intestinal feruloyl esterase

## Abstract

The purpose of this study was to determine whether the administration of the feruloyl esterase (FE)-producing strain *Lactobacillus fermentum* CRL1446 enhances metabolic and oxidative parameters in caloric-restricted (CR) mice. Balb/c male mice were divided into ad libitum fed Group (ALF Group), CR diet Group (CR Group) and CR diet plus *L. fermentum* Group (CR-Lf Group). CR diet was administered during 45 days and CRL1446 strain was given in the dose of 10^8^ cells/mL/day/mouse. FE activity was determined in intestinal mucosa and content at Day 1, 20 and 45. Triglyceride, total cholesterol, glucose, thiobarbituric acid reactive substances (TBARS) levels and glutathione reductase activity were determined in plasma. Gut microbiota was evaluated by high-throughput sequencing of 16S rRNA gene amplicons. At Day 45, total intestinal FE activity in CR-Lf Group was higher (*p* = 0.020) than in CR and ALF groups and an improvement in both metabolic (reductions in triglyceride (*p* = 0.0025), total cholesterol (*p* = 0.005) and glucose (*p* < 0.0001) levels) and oxidative (decrease of TBARS levels and increase of plasmatic glutathione reductase activity (*p* = 0.006)) parameters was observed, compared to ALF Group. CR diet increased abundance of Bacteroidetes and CRL1446 administration increased abundance of *Bifidobacterium* and *Lactobacillus* genus. *L. fermentun* CRL1446 exerted a bifidogenic effect under CR conditions.

## 1. Introduction

Caloric restriction (CR) is defined as a reduction in energy intake below the amount of calories that would be consumed ad libitum (≥10% in studies in humans and usually ≥20% in rodents) [1]. CR can be explained, from the point of view of evolution, as a compensation mechanism that is involved in metabolic and neuroendocrine responses that living organisms use to adapt to periods of food absence [2]. 

The general protocol used to perform CR experiments in animal models is to feed a group of animals ad libitum (control group), and another group with a lower percentage of daily ration than control group, whereby animals suffer a reduction in total caloric intake due to level of restriction [3]. CR has been regarded as the experimental regimen that can effectively lengthen lifespan in various animal models, but the actual mechanism remains controversial [4].

Gut microbiota has been shown to play a pivotal role in host health, and its composition is mostly influenced by diet [4]. Studies carried out on germ free mice demonstrated that these animals were resistant to diet-induced obesity, but gain weight upon transfer of gut microbiota from conventionally raised mice or ob/ob mice, potentially through increased capacity for energy harvest [5]. To date, there is no information regarding effect of caloric restriction diet in germ free mice.

Zhang et al. [4] reported that CR induced changes in gut microbiota can exert a health benefit to the host. Gut microbiota develops an intense metabolic activity that allows improving the bioavailability of nutrients and degradation of non-digestible compounds (through the contribution of enzymes or stimulation of endogenous enzymatic activities), the supply of new nutrients and the elimination of harmful anti-nutrients and other compounds. These metabolic functions have a major impact on health and nutritional status of the host; however, they depend on the composition of gut microbiota and its complex interactions with the diet and the individual [6].

Modulation of intestinal microbiota through probiotic bacteria administration is an important strategy to improve different nutritional states. In this field, bacteria with feruloyl esterase (FE) activity could be a promising option. The beneficial role of bacterial FE in human and animal health is due to their ability to increase the bioavailability of antioxidant compounds [7,8]. FE, also called cinnamoyl esterases (CE), are carboxyl ester hydrolases that hydrolyze hydroxycinnamate esters, with higher specificity on ferulic acid (FA). Hydroxycinnamates are commonly found in cereals, fruits and vegetables and are hydrolyzed by CE releasing hydroxycinnamic acids (HA) such as ferulic, sinapic, caffeic and *p*-coumaric acids [9]. FA induces intrinsic antioxidant mechanisms such as superoxide dismutase, catalase and glutathione reductase (GR) activities. Regular ingestion of this acid may provide substantial protection against oxidative stress-related ailments like cancer, diabetes, cardiovascular and neurodegenerative diseases, and in ageing [10]. 

Andreasen et al. [11] reported that intestinal CE activity has an epithelial and a microbial origin. This enzymatic activity is commonly found in different bacterial genera present in the human and rat gut [12], and levels and specificity of these enzymes are critical factors influencing the bioavailability of HA [13]. 

Recent studies reported that in mice fed with a normal diet, oral administration of *Lactobacillus fermentum* CRL1446, as a supplement or functional food, produced an increase in intestinal FE activity, enhancing the bioavailability of antioxidant FA, thus improving oxidative status [8,14]. Moreover, it has been reported that the administration of lactic acid bacteria (LAB) with FE activity can enhance metabolic activity markers in animal models of metabolic syndrome and diabetes [15,16].

Currently, there is no information regarding intestinal FE activity in animal models of caloric restriction and the effects of FE-producing LAB administration on metabolic and oxidative status of the host. Thus, the aim of this study was to determine whether the administration of feruloyl esterase-producing strain *L. fermentum* CRL1446 enhances metabolic and oxidative parameters in caloric restricted-mice.

## 2. Materials and Methods

### 2.1. Preparation of Bacterial Suspensions

*L. fermentum* CRL1446, strain isolated from an Argentinean goat milk cheese, was used in this study [17]. This strain was obtained from the Culture Collection of the Centro de Referencia para Lactobacilos (CERELA, Tucumán, Argentina). *L. fermentum* CRL1446 presented FE activity and was able to tolerate simulated gastrointestinal tract (GIT) conditions [18]. It was maintained in Man–Rogosa–Sharpe broth (MRS; Britania, Buenos Aires, Argentina) containing 20% (v/v) glycerol at −80 °C and propagated three times in MRS broth before each experiment.

For bacterial suspension preparation, *L. fermentum* CRL1446 was cultured in MRS broth for 16 h at 37 °C (late exponential phase), harvested by centrifugation (10,000× *g*, 10 min) and washed twice with phosphate buffered saline (PBS) pH 7.0. Cells were then resuspended in sterile drinking water to the desired concentration: 1 × 10^8^ cells per mL. Bacterial suspensions were prepared freshly every day and changed every 12 h. 

### 2.2. Animals and Diets

Recently weaned 21-day old male Balb/c mice (*n* = 84) were obtained from the closed random-bred colony maintained at CERELA. They were housed in individual cages and acclimated to 22 ± 2 °C with a 12 h light/dark cycle. They were then separated into the following three groups: ALF, ad libitum fed Group, CR, caloric restriction diet Group, CR-Lf, caloric restriction diet plus *L. fermentum* CRL1446 Group. ALF Group received normal diet (61% carbohydrates, 23% proteins, 7.5% fats, 4% raw fibre, 3.5%, total minerals (3.10 Kcal/g); Asociación de Cooperativas Argentinas, Buenos Aires, Argentina) and drinking water ad libitum for 45 days. This diet supplied approximately0.60 mg of hydroxycinnamates per day per mouse.

Animals from CR and CR-Lf Groups were acclimated for 5 days to ad libitum normal diet and after this period they were daily fed with a restricted diet (25% less than the daily ration) for 45 days in order to reach a degree of mild malnutrition (10%–25% body weight loss compared to mice from ALF Group). CR-Lf Group also received *L. fermentum* CRL1446 strain resuspended in the drinking water at the dose of 10^8^ cells/mL/day/mouse. In all groups, body weights were measured daily.

Twelve animals from ALF Group were sacrificed at Day 1 to evaluate the baseline values of intestinal FE activity (*n* = 6) and intestinal microbiota (*n* = 6). At Days 20 and 45 of feeding, 12 animals from each three groups were sacrificed for intestinal FE activity (*n* = 6) and microbiota (*n* = 6) determinations. Animals were fasted (12 h) before sacrifice. 

The experimental protocol was approved by the Animal Protection Committee of CERELA (CRL-BIOT-EF-2012/2A) and complied with current Argentinean laws.

### 2.3. Preparation of Intestinal Extracts

Small and large intestines were aseptically removed and different intestinal sections (SIM, small intestine mucosa; LIM, large intestine mucosa; SIC, small intestine content; LIC, large intestine content) were obtained according to Abeijón-Mukdsi et al. [8].

### 2.4. Determination of Intestinal FE Activity

Intestinal FE activity was determined in intestinal mucosa and content according to Abeijón-Mukdsi et al. [8]. Results were expressed as units (U) of FE activity per gram of intestinal mucosa or content. One unit was defined as the amount of enzyme releasing 1 mmol of ferulic acid per hour.

### 2.5. DNA Extraction from Intestinal Contents

Fifty milligrams of intestinal contents were homogenized in 500 µL of 10 mg/mL lysozyme solution in Tris-Sucrose buffer (50 mM Tris-HCl, 40 mM EDTA, 0.75 M sucrose, pH 8.0) and incubated for 1 h at 37 °C. Total DNA was purified using the Maxwell R 16 DNA Purification Kit and the Maxwell R 16 Instrument (Promega, Madison, WI, USA), according to the manufacturer’s instructions. 

### 2.6. High-Throughput Sequencing (HTS) of 16S rRNA Gene Amplicons

The bacterial V3-V4 16S rRNA regions were amplified by PCR using primer pairs 343F (5′-TACGGRAGGCAGCAG-3′) and 802R (5′-TACNVGGGTWTCTAATCC-3′) according to Polka et al. [19]. The PCR product pool was sent to Fasteris SA (Geneva, Switzerland) to be sequenced using the TruSeq™ DNA sample preparation kit (Illumina Inc., San Diego, CA, USA). High-throughput sequencing was carried out in one lane MiSeq Illumina instrument (Illumina Inc.) using the V2 chemistry in a 2 × 250 configuration sufficient to cover the entire amplicon length, estimated to be ~450 bp. Paired-end assembly and quality filtering was performed by using Flash software [20]. Sample de-multiplexing was carried out using DNA barcoding information per sample and Mothur platform. The assembled and barcode/primer-free sequences were processed for chimera removal using Uchime algorithm [21] and SILVA reference set of 16S sequences [22].

Diversity indexes were calculated with Mothur platform using default parameters and average method in the clustering step [23]. Different alpha diversity parameters such as the Observed (Sobs) and Chao’s richness, as well as the Shannon’s diversity and evenness indexes, which were computed from OTUs (operational taxonomic units) clustered at 97% and using a normalized subset of 23,000 sequences randomly selected after multiple shuffling (10,000×) of the original dataset. Taxonomy assignation at Phylum and Genus levels of the subset of high-quality reads was performed with Bayesian RDP Classifier [24], using an assignment confidence cutoff 0.8. Beta diversity was analyzed by performing Unifrac-weighted analysis [25].

### 2.7. Biochemical Assays

Blood was collected from all animals (*n* = 84) by cardiac puncture before sacrifice and transferred into tubes containing anticoagulant EDTA (Wiener Lab, Rosario, Argentina). Plasma was obtained by centrifugation (2500× *g*, 10 min), and used for biochemical assays, thiobarbituric acid-reactive substances (TBARS) assay and determination of glutathione reductase (GR) activity. Triglyceride, total cholesterol and glucose concentrations were measured by enzymatic methods using commercial kits (Wiener Lab, Rosario, Argentina). Protein concentrations were determined according to the method of Bradford [26], using a commercial kit (Bio-Rad, Hercules, CA, USA) and bovine serum albumin (Sigma, St Louis, MO, USA) as standard.

### 2.8. Lipoperoxidation and Glutathione Reductase (GR) Activity in Plasma

Lipid peroxidation was estimated spectrophotometrically by the thiobarbituic acid reactive substances (TBARS) method of Okawa et al. [27]. Results were expressed as nmol of TBARS per mg of protein, and presented as percentage, considering the basal TBARS levels in ALF Group as 100%.

GR activity was determined according to Esterbauer and Grill [28], by following the rate of NADPH oxidation at 340 nm. Results were expressed as units (U) of GR activity per mg of protein. One unit was defined as the amount of enzyme producing 1 nmol of oxidized NADP per minute.

### 2.9. Statistical Analysis

Results are means of three independent experiments ± standard error of the mean (SEM). Data were analyzed with one-way ANOVA using SPSS version 12.0 (SPSS Inc., Chicago, IL, USA), Tukey’s test was used to identify statistically significant differences (*p* < 0.05). 

## 3. Results

### 3.1. Effect of Caloric Restriction Diet and L. fermentum CRL1446 Administration on Body Weight

**Figure 1:**
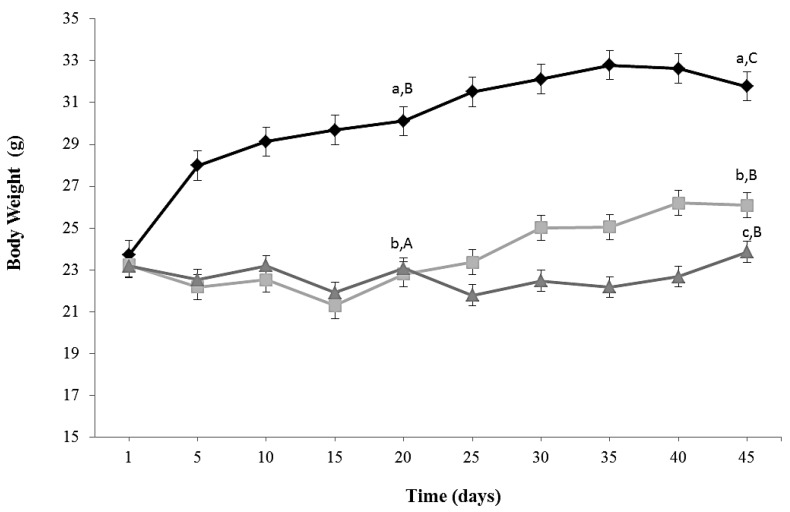
Body weight of ad libitum fed and caloric restricted mice: (♦) ALF: ad libitum fed Group; (■) CR: caloric restriction Group; and (▲) CR-Lf: caloric restriction plus *L. fermentum* CRL1446 administered Group. Data are means ± SEM (*n* = 12 per group). Data with different lowercase letters (a–c) at the same period of treatment and data with different uppercase letters (A–C) in the same group are significantly different (*p* < 0.05) according to ANOVA statistical analysis.

Kinetics of growth of the three experimental groups is shown in Figure 1. The protocol of caloric restriction diet induced a decrease in body weight of around 18% (CR and CR-Lf Groups) at Day 20 compared to ALF Group (*p* = 0.030). This decline in body weight was maintained until Day 45, observing a decrease of 24%–22% in CR and CR-Lf Groups, respectively, compared to ALF Group (*p* = 0.025). From Day 25 to Day 45, CR-Lf Group presented a body weight only 2% lower than CR group (*p* = 0.030).

### 3.2. Total Intestinal Feruloyl Esterase Activity in Mice

**Figure 2:**
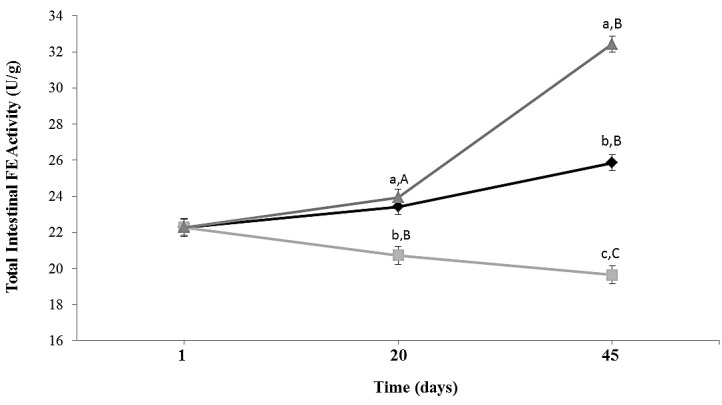
Total intestinal feruloyl esterase activity in ad libitum fed and caloric restricted mice: (♦) ALF: ad libitum fed Group; (■) CR: caloric restriction Group; and (▲) CR-Lf: caloric restriction plus *L. fermentum* CRL1446 administered Group. Data are means ± SEM (*n* = 6 per group at each time point). Data with different lowercase letters (a–c) at the same period of treatment and data with different uppercase letters (A–C) at same group are significantly different (*p* < 0.05) according to ANOVA statistical analysis.

Total intestinal esterase activity in ALF Group increased slightly during 45 days of feeding. In animals subjected to caloric restriction diet (CR Group), intestinal FE activity decreased 1.3 times (*p* = 0.0173) compared to ALF group at Day 45 (Figure 2). In CR-Lf Group total intestinal FE activity was similar at Day 20 and 1.25 times (*p* = 0.020) higher at Day 45 compared to ALF Group. CR-Lf Group showed a 1.65 times higher activity than CR Group at Day 45 (*p* = 0.008).

### 3.3. Feruloyl Esterase Activity in Different Intestinal Fractions

Feruloyl esterase activities in different intestinal fractions were determined at Day 20 and 45 (Table 1). In all groups, specific esterase activities in mucosa were 10–20 times higher than those detected in intestinal contents (*p* < 0.0001).

At Day 20 of feeding, statistically significant differences in esterase activity in LIC were observed. In this fraction, the activity was two times (*p* = 0.0010) and 1.47 times (*p* = 0.018) lower in CR and CR-Lf, respectively, than in ALF Group. A slight increase in feruloyl esterase activity in LIM of CR-Lf Group was observed (*p* = 0.035), compared to ALF and CR Groups (Table 1).

At Day 45, feruloyl esterase activity increased in all intestinal fractions from the three experimental groups compared to Day 20 (Table 1). SIM and LIM fractions activities of CR Group were lower than those detected in ALF (*p* = 0.032) and CR-Lf (*p* = 0.005) groups. No statistically significant differences in SIC activities of ALF and CR groups were observed. In CR-Lf Group, esterase activity of LIC fraction increased significantly compared to ALF and CR Groups (*p* = 0.0098). 

### 3.4. Effect of CR Diet and L. fermentum CRL1446 Administration on Intestinal Microbiota

The microbial communities present in mice fecal samples (*n* = 6) were analyzed by massive and parallel sequencing of 16S rDNA amplicons. Some alpha diversity parameters are depicted in Table 2. The observed richness (rarefaction analysis) showed that there were not strong differences among different gut microbiota obtained from all conditions. However, CR mice and especially those under CR-Lf treatment trended to decrease the number of theoretical gut microbial species when comparing with ALF mice. Interestingly, CR condition seemed to induce a gain of diversity in terms of community homogeneity. This was supported with data regarding the reciprocal Simpson’s index, where CR treatment at Day 45 almost doubled the diversity observed at Day 20, in contrast to that observed for the other two treatments.

**Figure 3:**
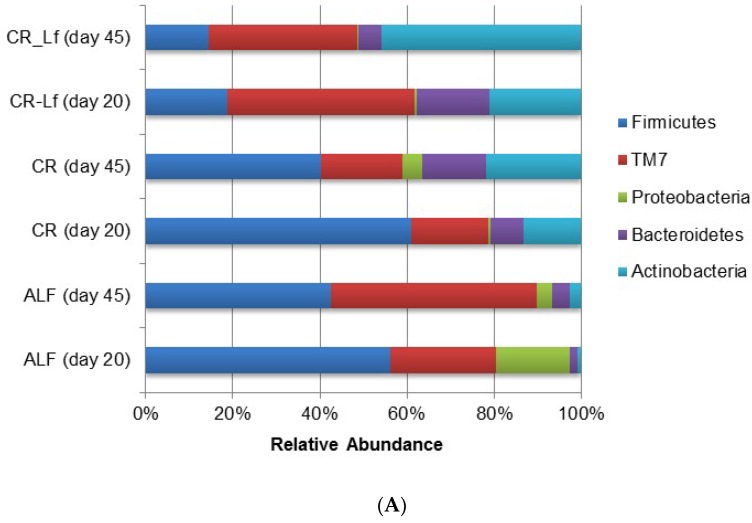

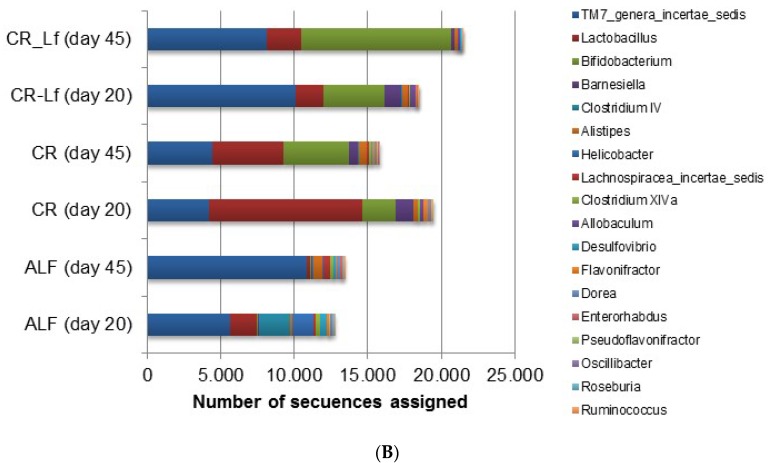
Relative abundance of gut bacterial communities in ad libitum fed and caloric restricted mice: (**A**) phylum level; and (**B**) genus level. ALF Group: ad libitum fed, CR Group: caloric restriction diet, CR-Lf Group: caloric restriction diet plus *L. fermentum* CRL1446.

The phylum and genus relative abundances of intestinal content microbiota are shown in Figure 3. Some important shifts in gut microbiota were observed among groups. Caloric restriction changed the relative abundance of certain bacterial groups at the phylum (Figure 3A) and genus (Figure 3B) levels. The effective number of sequences belonging to Firmicutes and retrieved from the ALF and CR groups accounted between 40% and 60% of the entire set of sequences analyzed. Accordingly, Firmicutes in the CR-Lf Group exhibited the lowest proportions in comparison with the other groups accounting for 18% and 14% of the microbial community at Day 20 and Day 45, respectively. CR-Lf Group was shown to be the only group with a positive fold-change in the ratio Firmicutes/Bacteroidetes between Day 20 and 45 of treatment (CR-Lf = 1.28).

In all experimental groups, an increase of Actinobacteria relative abundance was observed (Figure 3A) during the study. In CR-Lf Group, a pronounced increase of Actinobacteria in detriment of Bacteroidetes and even Firmicutes, was detected. *L. fermentum* CRL1446 administration allowed increasing Actinobacteria, reaching almost 50% of the relative abundance at Day 45 of treatment. 

When diversity was explored at genus level, a great difference in microbial community structure was observed between ALF and CR groups. Figure 3B shows the 18 most prevalent genera. In the analyzed samples, it was observed that almost 50% of the microbial community in ALF Group was represented by these 18 genera thus indicating a greater richness (Table 2). In the CR-Lf Group, three major genera (TM7, *Lactobacillus* and *Bifidobacterium*) accounted for almost 80% of diversity, which demonstrated a great dissimilarity in the community, as previously indicated by the Shannon’s Evenness index (Table 2). In CR group, a large proportion of *Lactobacillus* was observed comparable to the proportion of *Bifidobacterium*, detected in the CR-Lf Group. Totally, 0.98- and 1.30-fold changes in *Bifidobacterium* abundance were observed between Day 20 and Day 45 in CR and CR-Lf, respectively. From Day 20 to Day 45, *Lactobacillus* levels decreased in all groups, except in CRL-Lf (fold-change 45d/20d, ALF = −3.21; CR = −1.11; CR-Lf= 0.33).

### 3.5. Effect of CR Diet and L. fermentum CRL1446 Administration on Lipid Peroxidation and Glutathione Reductase Activity

**Figure 4:**
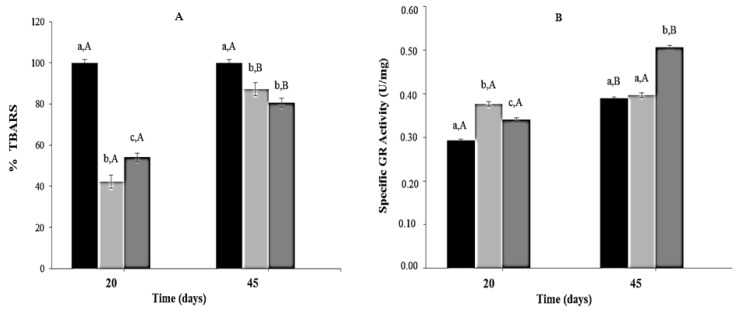
Lipid peroxidation (TBARS) and Glutathione Reductase (GR) activity in plasma of ad libitum fed and caloric restricted mice: (**A**) TBARS levels and (**B**) GR activity of: (■) ALF Group: ad libitum fed; (■) CR Group: caloric restriction; and (■) CR-Lf Group: caloric restriction plus *L. fermentum* CRL1446.Bars represent the mean ± SEM, *n* = 12 per group. Data with different lowercase letters (a–c) at the same period of treatment and data with different uppercase letters (A,B) in the same group are significantly different (*p* < 0.05) according to ANOVA statistical analysis.

Thiobarbituric acid reactive substances concentrations were significantly lower (40%–60%) in mice from CR and CR-Lf Groups at Day 20 of feeding compared to ALF Group (100%). At Day 45, significant differences in Thiobarbituric acid reactive substances concentration were observed among the three groups, the lowest levels being detected in CR-Lf (Figure 4A).

Regarding glutathione reductase activity, at Day 20, mice from CR and CR-Lf Groups showed a significant increase (~1.28 times, *p* = 0.005) compared to ALF Group. At Day 45, glutathione reductase activity in ALF Group increased 1.33times (*p* = 0.004) compared to Day 20. In this period, no significant statistical differences in glutathione reductase activity levels were observed between CR and ALF Groups; however, a significant activity increase (1.27 times, *p* = 0.006) was observed in CR-Lf Group (Figure 4B).

### 3.6. Effect of CR Diet and L. fermentum CRL1446 Administration on Biochemical Parameters

**Figure 5:**
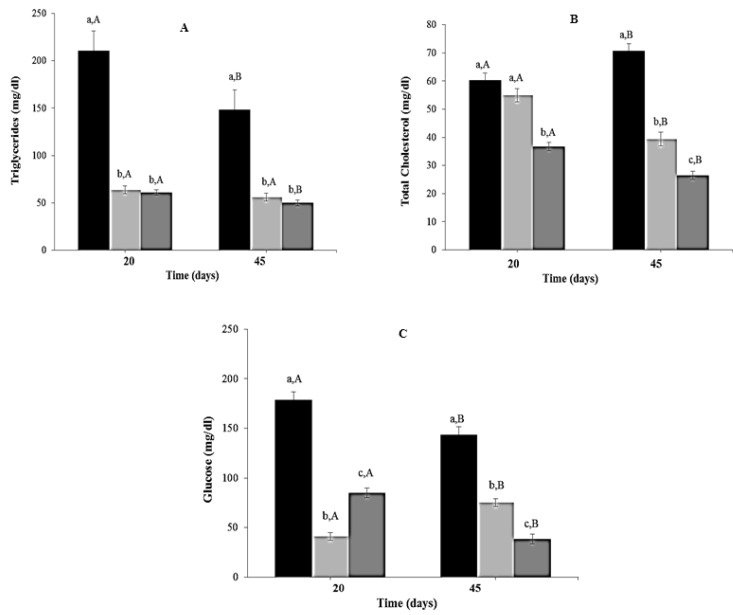
Biochemical parameters in plasma of ad libitum fed and caloric restricted mice, (■) ALF Group: ad libitum fed, (■) CR Group: caloric restriction, and (■) CR-Lf Group: caloric restriction plus *L. fermentum* CRL1446: (**A**) Triglycerides; (**B**) Total cholesterol; and (**C**) Glucose. Bars represent the mean ± SEM, *n* = 12 per group. Data with different lowercase letters (a–c) at the same period of treatment and data with different uppercase letters (A,B) in the same group are significantly different (*p* < 0.05) according to ANOVA statistical analysis.

Triglyceride levels decreased significantly in CR and CR-Lf Groups at Day 20 (3.31 to 3.46 times, *p* = 0.0012) and Day 45 (2.66 to 2.97 times, *p* = 0.0025), compared to ALF Group (Figure 5A). No significant differences were observed between these two treated groups. Total cholesterol levels at Day 20 were lower in CR-Lf Group (1.64 times, *p* = 0.011), compared to ALF and CR Groups (Figure 5B). No significant differences were observed between ALF and CR Groups at Day 20. However, at Day 45, total cholesterol levels decreased 1.80 times (*p* = 0.012) in CR and 2.67 times (*p* = 0.005) in CR-Lf Groups, compared to ALF Group. 

At both periods evaluated (Days 20 and 45), reductions in plasma glucose levels were observed in CR (*p* = 0.007) and CR-Lf Groups (*p* < 0.0001), compared to ALF Group (Figure 5C). The lowest levels were observed in animals from CR-Lf Group at Day 45 (38.56 mg/dL).

Regarding protein concentration, no significant differences were observed between the three experimental groups evaluated during the whole period of feeding (data not shown).

## 4. Discussion

There are many published epidemiological and clinical studies suggesting that CR decreases the incidence of cardiovascular disease, type 2 diabetes, and cancer in humans. CR without malnutrition slows the aging process and extends lifespan in diverse species by unknown mechanisms [29]. The inverse linear relationship between calorie intake and lifespan suggests that regulators of energy metabolism are involved in the effects of CR [30]. However, the evaluation of metabolic response to CR diet is a field that is not fully elucidated and requires further study. We evaluated the effects of a caloric restriction diet and *L. fermentum* CRL1446 administration, a probiotic strain, on changes of intestinal feruloyl esterase activity, microbiota, and metabolic and oxidative parameters in a mice model. CR diet administration during 45 days induced a 24% decrease in body weight (CR and CR-Lf Groups). The first degree malnutrition or mild malnutrition is characterized by a deficit of 10%–24% in body weight; second-degree malnutrition or moderate malnutrition, by a weight deficit of 25%–39%, and third degree malnutrition or severe malnutrition by a weight deficit of 40% or higher [31]. Thus, the experimental model used in this study corresponds to mild caloric restriction. Mice fed with CR diet plus *L. fermentum* CRL1446 presented a body weight 2% lower than CR Group from Day 25. Assays of CR diet administration were conducted in two different time periods (20 and 45 days) in order to establish the effects of feeding time with CR diet on intestinal FE activity, microbiota and biochemical and oxidative parameters in mice. Total intestinal esterase activity of ALF Group increased slightly during the 45 days of feeding, which could be due to stimulation of intestinal mucosa FE activity and/or induction of intestinal microbiota FE activity by dietary fiber as reported by Wang et al. [32]. In CR Group, lower levels of total intestinal FE activity were detected. *L. fermentum* CRL1446 administration to mice subjected to CR diet restored this activity to normal levels (similar to ALF Group) at Day 20, reaching the highest values at Day 45. In the three groups tested, mucosa esterase activities were 10 times higher than in intestinal contents. Similar results were reported in Swiss albino mice [8]. Other authors studied the distribution of esterase activity in Wistar rat intestine, reporting that it was located mostly in small intestine mucosa, while in large intestine, activity was detected predominantly in content [11]. At Day 45 of feeding, FE activity in intestinal mucosa increased in all experimental groups, and a 1.75- time increase in LIC from CR-Lf Group was observed. Two-time higher total intestinal FE activity was observed at Day 45 compared to Day 20, which was mainly due to an increase of intestinal content activities. These results were in accordance with the increased abundance of bacteria with FE activity detected in the CR-Lf group at Day 45. Intestinal FE activity has an epithelial and a microbial origin, contributing to hydrolysis of dietary hydroxycinnamates [11]. Abeijón–Mukdsi et al. [18] reported that *L. fermentum* CRL1446 was able to adhere to intestinal epithelial cells, suggesting that this strain can colonize the gut and increase intestinal FE activity.

Using high-throughput sequencing approaches to study changes in gut microbial communities, we found that gut microbiota was profoundly affected by caloric restriction. An increase of Bacteroidetes and Actinobacteria members in mice under CR diet (CR and CR-Lf groups) compared to ALF Group, was observed. Similarly, Ley et al. [33] observed that the proportion of Bacteroidetes increased with weight loss on two types of low-calorie diet. In CR-Lf Group, it was additionally found that the effective number of Bacteroidetes and Firmicutes was strongly reduced during treatment compared to CR Group. Among Actinobacteria, CR diet seems to promote the presence of mainly *Bifidobacterium* and *Lactobacillus* genus in the gut (Figure 3B) and *L. fermentum* administration accelerates the microbiota replacement increasing *Bifidobacterium* abundance. A bifidogenic effect was reported when *L. fermentum* CRL1446 was administered to normal diet fed mice [8]. 

On the other hand, Zhang et al. [4] reported that CR diet enriched the gut microbiota in phylotypes positively correlated with lifespan, for example, the genus *Lactobacillus*, and decreased those that were negatively correlated with lifespan, such as opportunistic pathogenic bacteria. Our results corroborate that CR diet increase the proportion of *Lactobacillus*, but only *L. fermentum* CRL1446 administration (CR-Lf Group) is able to maintain this genus during time. These results would confirm that CRL1446 strain colonizes the large intestine and thus increases FE activity in large intestine mucosa and content [18].

Competition between host and gut bacteria for nutrients may determine the composition of the feeding medium for homeostatic control of microbiota in the colon. Zhang et al. [4] suggested that, under conditions of restricted nutrient availability, as in CR and CR-Lf groups of our study, the host may extract nutrients (such as proteins and fats) more thoroughly, leaving primarily indigestible plant polysaccharides to the colon. This claim could explain the increased abundance of *Bifidobacteria* observed in CR and CR-Lf groups given the bifidogenic effect of dietary fiber [34]. Likewise, the increase in *Bifidobacteria* abundance could be the cause of the high levels of FE activity observed in LIC of CR-Lf mice, since FE activity was described in this genus [35].

To our knowledge, this is the first study reporting a bifidogenic effect associated to caloric restriction diet. Although the molecular basis of such effect remains elusive to determine if this is derived from availability of dietary fiber in colon or maybe caused by host metabolic byproducts. CR remains as a cost-effective treatment to promote the growth of such beneficial bacteria. *Bifidobacteria* can improve the gut barrier function and short-chain fatty acid production in the gut to reduce the risk of infections by opportunistic pathogens [36] as well as promote longevity by modulating apoptotic signals inside the cell [37].

In our experimental protocol, animals were fed with a conventional balanced diet containing 3.40% fiber, mostly from corn, which is rich in hydroxycinnamates. In cereals, the most abundant hidroxycinnamic acid is *trans*-ferulic acid, which is found mainly as ester-linked to arabinofuranose containing polysaccharides. Hydroxycinnamic acid exhibit antioxidant properties in vitro [38], and low doses of ferulic acid, mostly, have been linked to the prevention of oxidative stress and lipid peroxidation [10]. To determine whether the increase in intestinal feruloyl esterase activity observed in mice fed with *L. fermentum* CRL1446 could be related to some improvement in the oxidative status of the animals, we determined levels of TBARS and glutathione reductase activity in the plasma of mice from different groups (ALF, CR and CR-Lf). Abeijón-Mukdsi et al. [8] reported that oral administration of *L. fermentum* CRL1446 decreased basal TBARS percentage (30%–40%) after 5 days of treatment in ad libitum fed Swiss albino mice. Bruss et al. [39] reported that in CR there is a decrease in the oxidation and synthesis of fatty acids, which is an important metabolic adaptation to nutritional stress. In this regard, our study revealed that a short-term CR (20 days) had a beneficial effect in mice by reducing the percentage of TBARS in plasma. At Day 45, a significant increase in glutathione reductase activity was observed in CR-Lf Group. Our results demonstrate that administration of *L. fermentum* CRL1446 increases intestinal feruloyl esterase activity, thus the hydrolysis of dietary hydroxycinnamates and the bioavailability of ferulic acid in the gut. This acid may be absorbed at intestinal level, stimulating glutathione reductase activity. Skrha [40] reported that CR diet can accelerate antioxidant response mechanisms by gene expression or activation of antioxidant enzymes. Increase in glutathione reductase activity indicates a stimulation of this enzyme, which is important because of its involvement in the regeneration of reduced glutathione (GSH), which is extremely useful for the endogenous antioxidant defense. The increase in glutathione reductase activity after 45 days of CR diet administration in CR-Lf Group highlights the importance of the administration of *L. fermentum* CRL1446 strain, which would offer additional protection against oxidative stress. 

CR diet promotes improved lipid profile (decreased plasmatic triglyceride and total cholesterol) and it lowers blood glucose level [41]. In our study we observed that triglyceride levels decreased significantly in CR and CR-Lf Groups at Days 20 and 45 of CR diet administration compared to ALF Group. These results indicate that diet is the sole responsible for the decrease of triglyceride levels, and not the administration of *L. fermentum* CRL1446. The duration of the administration period of CR diet influences serum total cholesterol levels. Compared to ALF Group, total cholesterol levels decreased at Day 20 in CR-Lf Group, but not in CR-Group. These results indicate that CR diet is not responsible for changes in total cholesterol levels in this period. At Day 45, total cholesterol levels were lower in the groups that received the CR diet with and without bacteria administration, the lowest mean values being observed in CR-Lf Group. These results suggest that CRL1446 strain has a hypocholesterolemic effect in the host. It is also important to note that the duration of the administration period of CR diet influences serum total cholesterol levels. Bhathena et al. [7] reported that a microencapsulated FE-producing *Lactobacillus fermentum* LF11976 probiotic formulation lowers serum lipids in hypercholesterolemic hamsters. Ferulic acid presents hypocholesterolemic properties since it can inhibit the hydroxymethylglutaryl-CoA reductase, the rate-limiting enzyme in cholesterol biosynthesis, and cholesterol acyltransferase, the cholesterol-esterifying enzyme in tissues, and by increasing the acidic sterol excretion [42]. 

CR diet decreased glucose levels at both Day 20 and 45 of treatment. Interestingly, at Day 45, higher glucose levels than at Day 20, were observed. This behavior may be due to the fact that in the CR in mammals there are two phases, an adaptive period and a steady state period. During the adaptive phase, glucose metabolism regulates secretion of insulin from the pancreatic cells. In the immediate response to low levels of glucose, the animal quickly degrades glycogen stores upon the secretion of glucagon. The liver assumes a dominant role during CR by up-regulating the expression of enzymes involved in gluconeogenesis and down-regulating those in glycolysis. Thus, blood glucose falls precipitously during the adaptive period, and rises to a higher, but still below normal level during the steady state [43].

In our study, the lowest glucose levels were observed in CR-Lf Group at Day 45. *L. fermentum* CRL1446 could regulate blood glucose levels by stimulating insulin secretion and promoting the survival of β-cells of the pancreas through its feruloyl esterase activity. Adisakwattana et al. [44] reported that ferulic acid may improve glucose tolerance, lipid metabolism, body weight and other metabolic markers. 

The mechanism by which diet modulation influences intestinal microbiota and, concomitantly, host health requires further elucidation. 

## 5. Conclusions

This study demonstrates that caloric intake, extent of treatment (20–45 days) and FE-producing lactic acid bacteria administration produce changes in intestinal feruloyl esterase activity, microbiota profile, metabolic and oxidative parameters. Overall, these results show that feeding mice with a CR diet increased relative abundance of beneficial bacteria such as *Lactobacillus* and *Bifidobacterium* and improved metabolic and oxidative parameters at Day 45, compared to ALF Group. Administration of *L. fermentum* CRL1446 increased relative abundance of Bifidobacteria and was able to maintain *Lactobacillus* genus during the time of treatment, increasing intestinal feruloyl esterase. Moreover, this strain enhanced the beneficial effect of CR due to its hypocholesterolemic and hypoglycemic properties, being its effects evident even at Day 20 of treatment. *L. fermentum* CRL1446 could be used as a complementary therapeutic of diseases associated to hypercholesterolemia and hyperglycemia.

## Figures and Tables

**Table 1:** Feruloyl esterase activity in different intestinal fractions at 20 and 45 day of feeding.

Source of Enzyme	Day	Groups		
		ALF	CR	CR-Lf
SIM	20	11.66 ± 0.06 ^a^	11.12 ± 0.08 ^a^	11.35 ± 0.09 ^a^
	45	14.60± 0.07 ^b^	11.83 ± 0.53 ^c^	15.29 ± 0.04 ^a^
LIM	20	10.20 ± 0.06 ^b^	8.29 ± 0.05 ^c^	11.05 ± 0.07 ^a^
	45	14.21 ± 0.04 ^b^	12.99± 0.06 ^c^	14.40 ± 0.05 ^a^
SIC	20	0.84 ± 0.05 ^a^	0.88 ± 0.08 ^a^	0.87 ± 0.04 ^a^
	45	1.28 ± 0.06 ^a^	1.29 ± 0.07 ^a^	1.26 ± 0.06 ^a^
LIC	20	0.91 ± 0.06 ^a^	0.47 ± 0.02 ^c^	0.62 ± 0.04 ^b^
	45	1.14 ± 0.07 ^b^	0.85 ± 0.05 ^c^	1.49 ± 0.05 ^a^

Values are expressed as the mean ± SEM, *n* = 6 per group. Values with different superscript letters in the same row are significantly different (*p* < 0.05) as assessed by one-way ANOVA. ALF, ad libitum fed Group; CR, caloric restriction diet Group; CR-Lf, caloric restriction diet plus *L. fermentum* CRL1446 administered Group; SIM, small intestine mucosa; LIM, large intestine mucosa; SIC, small intestine content; LIC, large intestine content.

**Table 2:** Common ecological parameters of alpha diversity for the microbial communities analyzed.

Sample	Distance	Sobs	Chao’s Richness Index	Shannon Diversity Index	Simpson Index	Shannon Evenness Index
ALF20	0.03	1374	10,684	3.677	0.083	0.508
ALF45	0.03	1400	8550	3.477	0.196	0.479
CR20	0.03	1275	10,536	3.022	0.160	0.422
CR45	0.03	1399	11,768	3.628	0.084	0.500
CR-Lf20	0.03	1269	9183	2.771	0.204	0.387
CR-Lf45	0.03	1193	12,337	2.219	0.274	0.313

ALF20, *ad libitum* fed day 20 Group; ALF45, *ad libitum* fed day 45 Group; CR20, caloric restriction diet day 20 Group; CR45, caloric restriction diet day 45 Group; CR-Lf20, caloric restriction diet plus *L. fermentum* CRL1446 administered day 20 Group; CR-Lf45, caloric restriction diet plus *L. fermentum* CRL1446 administered day 45 Group.
